# Determinants of mother to child HIV transmission (HIV MTCT); a case control study in governmental health centers of East Gojjam Zone, Northwest Ethiopia, 2019

**DOI:** 10.1186/s12978-022-01501-y

**Published:** 2022-09-29

**Authors:** Mengistie Kassahun Tariku

**Affiliations:** Department of Public Health, College of Health Science, Debre Markos University, Debre Markos, Ethiopia

**Keywords:** HIV positive mothers, Prevention of mother to child transmission and HIV exposed infants

## Abstract

**Background:**

Mother to child human immune virus (HIV) transmission is the passage of HIV from mother to her child during pregnancy, labor, delivery or breast-feeding. The objective of this study was to identify determinants of mother to child HIV transmission among HIV exposed infants who were born from HIV positive mothers at Governmental health centers of East Gojjam Zone, Northwestern Ethiopia, 2019.

**Methods:**

A case–control study was conducted on 210(42 cases and 168 controls) from April 1 to 30/ 2019. All cases were included in the study. Controls were selected by simple random sampling. Secondary data were extracted by using checklists from the document of 8 health centers ART register book, antenatal care (ANC) follow up register book, PMTCT service registration log book charts and infant dried blood sample (DBS) tally sheets. After Bivariable logistic regression analysis, all variables with p-value $\le$ 0.25 were entered into multivariable logistic regression and p value < 0.05 considered as significantly associated with the outcome variable.

**Results:**

HIV exposed infants who were being rural dweller versus (vs) those infants who were being urban dweller [adjusted odds ratio (AOR) = 3.73; 95% CI; 1.27–10.69], have no history of antenatal care follow up of HIV exposed infants’ HIV positive mothers versus (vs) those mothers who have been having history of ANC follow up [ AOR = 5.0;95%CI; 2.02–12.16] and initial CD4 count of HIV infants’ mothers $\le 350$ vs those mothers whose CD4 > 350 [AOR = 2.7;95%CI;1.35–5.52] were significantly associated with HIV infection.

**Conclusion:**

Mother to child HIV transmission was significantly associated with history of ANC follow up of exposed infants’ mothers and initial CD4 counts of mothers. Strong effort should be made to further increase the ANC service utilization of HIV positive pregnant women.

## Plain English summary

Prevention of Mother to child human immune virus (HIV) transmission is the primary method of infection among children. To eliminate infection in children and keep mothers alive, a comprehensive package of interventions accelerating, including preventing of women from becoming infected with HIV, protect unwanted pregnancy, throughout pregnancy, provide skilled delivery, exclusive breastfeeding and providing appropriate HIV treatment, care, and support for mother and infants. Despite the availability and scale-up of life-saving interventions, only a few pregnant women living with HIV in Ethiopia have been receiving the medication to prevent mother to child HIV transmission. There is limited information about determinant of mother to child HIV transmission in Ethiopia.

In this study, the data were extracted from the document of health centers ART register book, ANC follow up register book, PMTCT service registration log book charts and infant DBS tally sheets in East Gojjam, Ethiopia.

This study revealed that high proportion of HIV infection among exposed infants in the study area. Infections with HIV among HIV exposed infants were positively associated with history of ANC follow up of their mothers and initial CD4 counts of their mothers.

## Introduction

Prevention of Mother to child human immune virus (HIV) transmission is the passage of HIV from mother to her child. It is also the primary method of infection among children [1]. This transmission will occur during pregnancy, labor and delivery, or breastfeeding. Without intervention, about 15–30% of babies born to HIV positive women will be infected with HIV during pregnancy and at birth in the world. A further 5–20% of 18–24 months’ children will become infected through breastfeeding [2, 3]. Globally, one million HIV exposed infants born with HIV infected women every year [1, 4]. There are approximately 1.4 million HIV positive women who become pregnant and contribute to more than 300,000 neonatal and fetal deaths each year in the world [5]. According to world health organization 2010 report, the prevalence of HIV in infants who are born from HIV positive mothers they attend both on treatment and prophylaxis was 10.9% in the world [6]. Globally, 3.2 million Children living with HIV are 91% live in sub-Saharan Africa 6% living Asia and Pacific; the remaining 3% are situated in the rests of the world [7, 8].

These burdens account especially 22 countries, from these Ethiopia is one of the priority countries where one of every 3 children born from women living with HIV. The Government has been accelerating to expand the prevention of mother to child transmission (PMTCT) of HIV service by endorsing with antenatal care free of charge [9]. To eliminate infection in children and keep mothers alive, a comprehensive package of interventions accelerating, including preventing of women from becoming infected with HIV, protect unwanted pregnancy, throughout pregnancy, provide skilled delivery, exclusive breastfeeding and providing appropriate HIV treatment, care, and support for mother and infants [10]. Moreover, in 2013, all pregnant women have been considered eligible to start long-term antiretroviral therapy (ART) through a package is called option B + which has a great role to ensure prevention of at least 98% of mother to child transmission of HIV (PMTCT) [11].

Infants are infected with HIV at least 1600 every day and more than 600,000 infants are infected by the virus annually mostly in developing countries mainly in sub-Saharan Africa [12]. In Ethiopia, an estimated 1.2% of pregnant women are living with HIV consequently one of every three children born to this woman is being infected with HIV [7].

In Ethiopia, despite the availability and scale-up of life-saving interventions, only 24% of 13,000 pregnant women living with HIV have been receiving the medication to prevent MTCT of HIV even women who utilize skilled delivery services are 12% [13]. The aim of this study was to identify determinants of mother to child HIV transmission among HIV exposed infants who were born from HIV positive mothers at Governmental health centers of East Gojjam Zone, Northwestern Ethiopia.

## Methods

### Study setting and period

Institution based case–control study design was conducted from April 1 to 30/ 2019 in 8 government health centers of East Gojjam Zone, Northwest Ethiopia. The 2017 data shows that in regional health bureau numbers of pregnant women tested and know their results during pregnancy, labor, delivery and post-partum period is 452,566. In third quarter of 2017 annual report was number of HIV exposed infants who were receiving ARV prophylaxis were 2,019 and HIV confirmatory (antibody test) by 18 months were 2122 among these 58 were HIV positive. In our study area, the total population was 2,632,632 among these the reproductive age women were 63,000 [14].

Sample size was computed by using Epi Info version 7 with double population proportion formula. It was calculated with the assumption of infant feeding practice as a factor [15], 95% confidence interval, 80% power, 10% non-response rate, and case to control ratio 1:4 to increase the power of the study. The total calculated sample size was 105 (21 cases and 84 controls). There was no any need of sampling from 42 cases. So, all HIV positive infants were included in the study. Controls were selected by simple random sampling. Therefore, the total sample size was 210 (42 cases and 168 controls).

Structural extracted data tools that consisted of socio-demographic, clinical, obstetric and infant related variables. Before data collection, data collectors were trained for half of a day about how data were collected. Pre-test was also done in 5% of the samples to cheek the quality of questioners from other than our samples. In our study, the data were extracted from the document of 8 health centers’ ART register book, ANC follow up register book, PMTCT service registration log book charts and infant DBS tally sheets. Our data assurance was closely followed by both the Supervisor and data collectors that was contribute for quality of the study. Maintenance of privacy and confidentiality of the data was secured. Every day all collected data were reviewed and checked at the end of data–collection and any error was corrected. Daily checking of missing values and incomplete data was done in order to enhance the quality of data.

The data were collected from 8 health centers that provide PMTCT Services and the data were clean and edit before entry and checking for correction. Collected data were entered by using Epi data version 3.1 and then it was exported to SPSS. The exported data was analyzed and presented by using descriptive summary statistics and tables. After bivariate logistic regression analysis, all variables with a p-value less than or equal to 0.25 was entered into multivariable logistic regression and p value < 0.05 considered as significantly associated with the outcome variable.

## Results

In this study, the total participants were 210 (42 cases and 168 controls). More than half 22 (52.4%) of cases and 90(53.6%) of controls were male. Fourteen (33.3%) of cases’ mother of educational level was primary, and 46 (27.4%) of controls’ mother of educational level was secondary. About 32(76.2%) of cases and 137(81.5%) of controls were urban dwellers. Nearly three-fourth, 31(73.8%) of cases, and 151 (89.9%) of controls whose mothers had history of antenatal care follow up. Majority, 34 (81.0%) of cases’ father and 161(95.8%) of controls’ father have been tested for HIV. More than half 22(52.4%) of cases’ mothers’ and controls mothers’121 (72.0%) of CD4 level was greater than 350 (Table 1).

**Table 1:** Characteristics of study participants in East Gojjam Zone, Northwest Ethiopia, 2019

Variables		Respondent status	
		Cases (n) = 42	Controls(n) = 168
		N (%)	N (%)
Sex of the infant	Male	22 (52.4)	90 (53.6)
	Female	20 (47.6)	78 (46.4)
Level of education	Unable to read and write	9 (21.4)	16 (9.5)
	Read and write but have no formal Education	3 (7.1)	26 (15.5)
	Primary	14 (33.3)	37 (22.0)
	Secondary	8 (19.0)	46 (27.4)
	Above secondary	8 (19.0)	43 (25.6)
Residence	Urban	32 (76.2)	137 (81.5)
	Rural	10 (23.8)	31 (18.5)
Did the mother attend antenatal	Yes	31 (73.8)	151 (89.9)
	No	11 (26.2)	17 (10.1)
Partner tested	Yes	34 (81.0)	161 (95.8)
	No	8 (19.0)	7 (4.2)
Place of delivery	Health facility	38 (90.5)	161 (95.8)
	Home	4 (9.5)	7 (4.2)
Mode of delivery	Spontaneous vaginal delivery	34 (81.0)	145 (86.3)
	Caesarean section	7 (16.7)	16 (9.5)
	Others	1 (2.4)	7 (4.2)
First 6 month’s feeding	Exclusive breast feeding	36 (85.7)	146 (86.9)
	Mixed feeding	6 (14.3)	22 (13.1)
Did the mothers Had ARV intervention	Yes	31 (73.8)	167 (99.4)
	No	11 (26.2)	1 (0.6)
Did child receive ARV prophylaxis	Yes	42 (100.0)	166 (98.8)
	No	0 (0.0)	2 (1.2)
Is the infant start CPT	Yes	40 (95.2)	163 (97.0)
	No	2 (4.8)	5 (3.0)
WHO clinical stage near/at delivery	Stage 1	23 (54.8)	110 (65.5)
	Stage 2	9 (21.4)	22 (13.1)
	Stage 3	8 (19.0)	29 (17.3)
	Stage 4	2 (4.8)	7 (4.2)
Maternal CPT started	Yes	29 (69.0)	131 (78.0)
	No	13 (31.0)	37 (22.0)
Mothers age group	< 25 year	14 (33.3)	37 (22.0)
	25–35	23 (54.8)	82 (48.8)
	> 35	5 (11.9)	49 (29.2)
Weight of new borne	< 2500 g	9 (21.4)	30 (17.9)
	≥ 2500	33 (78.6)	138 (82.1)
Age enrolment	≤ 6 week	9 (21.4)	49 (29.2)
	> 6 week	33 (78.6)	119 (70.8)
Age start CPT	≤ 6 week	9 (22.5)	14 (8.6)
	> 6 week	31 (77.5)	149 (91.4)
CD4cat	> 350	22 (52.4)	121 (72.0)
	≤ 350	20 (47.6)	47 (28.0)

In Bivariable analysis educational level of mothers, residence, ANC follow up, History of infant IPT taking and CD4 level of mothers were significantly associated with HIV infection for exposed infants and included in multivariable analysis.

In Multivariate logistic regression analysis, residence, ANC follow up and CD4 count for mothers were significantly associated with HIV infection for exposed infants (Table 2).

**Table 2:** Multivariate logistic regression analysis of factors associated with HIV infection for exposed infants at governmental health centers in East Gojjam Zone, Amhara region, Ethiopia, 2019

Variables		Respondents status		COR (95%CI)	AOR (95%CI)	P-value
		Cases	Controls			
Education level	Unable to read and write	9	16	1	1	0.087
	Read and write no formal education	3	26	3.0 (0.76–11.90)	0.3(0.10–0.97)	0.045
	Primary	14	37	1.3 (0.51–3.19)	1.2(0.28–5.24)	0.798
	Secondary	8	46	3.1 (1.12–8.49)	0.5(0.17–1.24)	0.124
	Above secondary	8	43	3.2 (1.15–8.80)	1.2(0.39–3.42)	0.788
Residence	Urban	32	334	1	1	0.001
	Rural	10	47	2.2 (1.04–4.81)	3.73(1.27–10.69)	
ANC	Yes	31	352	1	1	0.000
	No	11	29	4.3 (2.06–9.44)	5.0 (2.02–12.16)	
Infant CPT	Yes	40	375	1	1	0.149
	No	2	6	0.3 (0.06–1.64)	0.2 (0.03–1.68)	
CD4 Count	> 350	22	255	1	1	0.004
	$\le$ 350	20	126	1.84 (1.04–5.85)	2.7 (1.35–5.52)	

## Discussion

The main objective of this study was to identify determinants of mother to child HIV transmission among HIV exposed infants who were born from HIV positive mothers at Governmental health centers of East Gojjam Zone, Northwestern Ethiopia. Residence was significantly associated with MTCT of HIV. HIV exposed infants who live in rural were almost 4 times more likely contract HIV than HIV exposed infants who live in urban. This might be due to poor accessibility of PMTCT in rural area.

HIV exposed infants’ mother who had not ANC follow up almost 5 times more likely their infants could be infected by HIV than who had ANC follow up. This might be due to ANC follow up will enable to provide PMTCT intervention which includes early identification of HIV positive mothers and initiation of ARV drugs. This is similar with the study conducted in Wello Dessie [16]. But this is discrepancy with the study done in Brazil [17], Jima Ethiopia [18] and Bishoftu hospital Ethiopia [19]. This discrepancy might be the result of integration of the PMTCT interventions with ANC services in option B+.

HIV exposed infants who born from mothers whose CD4 count $\le$ 350 is almost 2.7 times more infected by HIV than infants born from mothers’ CD4 count $<$ 350. This might be due to higher viral load which increase HIV transmission from mother to infant. There might be also a genital ulcer which increases the risk of MTCT of HIV [20].

## Conclusion

Infections with HIV among HIV exposed infants were positively associated with residence, history of ANC follow up of their mothers and initial CD4 counts. Further effort is needed to address ARV intervention to all HIV positive pregnant women to improve CD4 count of HIV positive pregnant women.

Strong effort should be made to further increase the ANC service utilization of HIV positive pregnant women with the help of city health extension workers and mother support groups.

### Limitation of the study

This study did not generalize for all HIV exposed infants for whom mothers did not have health facility visit.

## Data Availability

The data sets generated during the current study are available from corresponding author on reasonable request.

## Abbreviations

AIDS: Acquired immune deficiency syndrome
ANC: Antenatal care
ART: Antiretroviral treatment
DBS: Dried blood spot
AOR: Adjusted odd ratio
HIV: Human immune virus
CI: Confidence interval
PMTCT: Prevention of mother to child transmission
